# Femoral offset restoration affects the early outcome of revision in patients with periprosthetic femoral fractures of Vancouver B2 - a single-center retrospective cohort study

**DOI:** 10.1186/s12891-023-06694-2

**Published:** 2023-07-11

**Authors:** Lei Sun, Wenjing Song, Zhongyuan Zhang, Ziyao Xu, Mengshuai Sun, Guangling Gao, Hongjiang Jiang, Changjun Ju

**Affiliations:** Department of Orthopaedics, Wendeng Orthopaedic Hospital of Shandong Province, Weihai, 264400 China

**Keywords:** Femoral offset, Periprosthetic femoral fractures, Revision, Tapered fluted modular titanium stem

## Abstract

**Background:**

Femoral offset (FO) restoration plays an important role in improving the prognosis and quality of life of patients undergoing hip replacement. However, it is not given enough attention in revisions among patients with periprosthetic femoral fractures (PPFFs); instead, more attention is given to reduction, fixation of fractures and stabilizing prostheses. The primary objective of this study was to evaluate the effect of FO restoration on the function of the hip joint in revisions of patients with PPFF of Vancouver B2. Moreover, we studied whether there is a difference in FO restoration between modular and nonmodular stems.

**Methods:**

A retrospective review of 20 patients with PPFF of Vancouver B2 revised with a tapered fluted modular titanium stem and 22 patients with PPFF of Vancouver B2 revised with a tapered fluted nonmodular titanium stem from 2016 to 2021 was conducted. Based on the difference between the FO of the affected side and that of the healthy side, 26 patients were allocated into Group A (difference ≤ 4 mm), and 16 patients were allocated into Group B (difference > 4 mm). The postoperative Harris Hip Score (HHS), range of motion of the hip joint, length of both lower limbs and dislocation were compared between Group A and Group B. The proportions of patients with FO restoration (difference ≤ 4 mm) and stem subsidence were compared between the modular and nonmodular groups.

**Results:**

The mean follow-up time was 34.3 ± 17.3 months, and all cases achieved fracture healing at the last visit. Patients in Group A had a higher HHS, larger range of abduction, fewer dislocations and less limb length discrepancy (LLD). Patients in the modular group had a higher proportion of FO restoration and less subsidence.

**Conclusion:**

FO restoration improves postoperative hip joint function and reduces dislocation and LLD in revisions of patients with PPFF of Vancouver B2. Compared with nonmodular prostheses, modular prostheses tend to be easier for FO restoration under complex circumstances.

## Background

The incidence of periprosthetic fractures after primary total hip arthroplasty (THA) is 1%~2.5%, while the incidence after THA revision is as high as 4-12% [1]. Most of the fractures are periprosthetic femoral fractures (PPFFs). Periprosthetic fractures account for 9.3–14.7% of all THA revisions, second only to aseptic loosening of the prosthesis and infection [2]. As the number of patients undergoing THA increases, periprosthetic fractures are becoming more common. According to the Vancouver classification system [3], patients with Vancouver B2 have loose prostheses and no significant loss of bone volume. Treatment goals for Vancouver B2 fractures include restoring the long-term stability of the femoral prosthesis and achieving fracture healing [4]. Treatment with internal fixation in type B2 fractures leads to a significantly longer healing time and lower mobility than revision [5], so revisions are good choices for most cases.

Femoral offset (FO) is the distance from the center of rotation of the femoral head to a line bisecting the long axis of the femur, which varies with the center of rotation of the femoral head and the size of femur [6]. FO affects the abductor lever arm and maintains the balance between gravity and the tension of the abductor muscles [7, 8]. Insufficient or excessive FO after THA incurs a series of accompanying problems, such as imbalance of soft tissue tension, instability of the hip joint and dislocation, thus affecting the function of the hip joint [9]. Postoperative global FO > 5 mm less than that of the contralateral hip after THA was associated with hip abductor muscle weakness, resulting in gait asymmetry [10].

Although restoration of the FO has become a consensus in THA, we found that it is not commonly achieved in revisions of patients with VB2 based on our clinical observations. As a result, some patients had postoperative LLD, dislocation or hip joint dysfunction, but there were few studies on FO restoration in revisions of patients with VB2. Compared with restoring the stability of the femoral prosthesis and fracture healing, restoration of FO is underappreciated. In this retrospective study, we investigate the importance of FO restoration for hip joint function in revisions of patients with VB2. Furthermore, we investigated the effect of prosthesis selection on FO restoration.

## Methods

### Study design

We retrospectively reviewed the medical records of 42 patients who met the inclusion and exclusion criteria and were treated at the Department of Orthopedics, Wendeng Orthopedic Hospital of Shandong Province between January 2016 and April 2021. This study was approved by the Ethics Committee of the Hospital, and all patients provided informed consent to be included in this study.

### Patient information

Patients undergoing revision with PPFF of VB2 were screened in this study. The study inclusion criteria were as follows: 1) ≥ 50 years old; 2) fractures caused by low-energy injuries; and 3) ASA (American Society of Anesthesiologists) classification less than III. The exclusion criteria were as follows: (1) pathologic fractures; (2) walking difficulties before fracture; (3) patients who received conservative treatment or only open reduction and internal fixation; and (4) patients who were unable to follow-up over an 18-month period.

### Surgical procedures

**Fig. 1:**
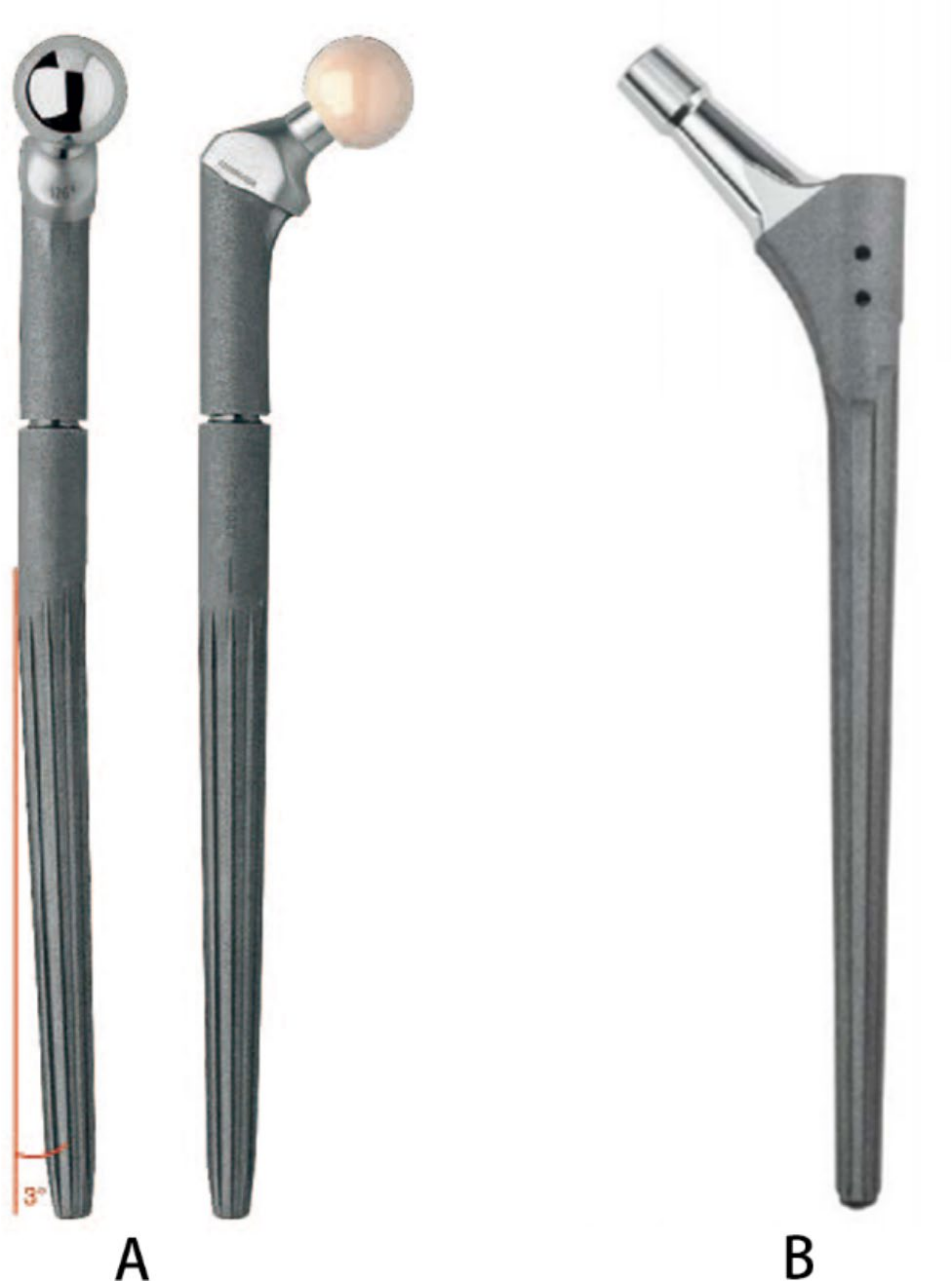
**A**: Tapered fluted modular titanium stem (MP; Waldemar Link, Hamburg, Germany). The dentate structure in the proximal part of the prosthetic stem can bind with the tooth in the head and neck component. These two parts are fixed by screws. The surface of the prosthesis is covered with micropores of 70 microns, which is good for bone ingrowth. The distal end of the stem is tapered with a 3-degree curvature, which helps accommodate the bow of the patient’s femur. The prominent crests on the surface increase the fixation of the distal femur. **B**: tapered fluted nonmodular titanium stem (WE-Cone; WeiGao, Weihai, China). The distal end of the stem is tapered with 8 crests to increase its anti-rotational stability

All operations were performed by two senior surgeons under spinal anesthesia or general anesthesia. Patients were placed in the lateral position, and the posterolateral extension approach was used. After hip joint dislocation, surgeons tested the stability of the femoral stem by longitudinal traction. After confirming the loosening of the prosthesis, the femoral stem was removed. Based on the evaluation of the patient’s preoperative X-ray and intraoperative testing, a tapered fluted modular titanium stem (MP; Waldemar Link, Hamburg, Germany) or a tapered fluted nonmodular titanium stem (Wagner SL; Zimmer, Warsaw, IN or WE-Cone; WeiGao, Weihai, China), as Fig. 1 shows, was implanted at least 5 cm below the fracture [11]. Then, the fracture was reduced and fixed by wires and cables. In the modular group, surgeons can adjust the length of the femoral stem by choosing head and neck components of different lengths or using gaskets, and FO is adjusted by choosing head and neck components of different FO and changing the anteversion angle. After the femoral head model was implanted, the hip joint was reduced, and the stability of the hip joint was tested. After ensuring stability, the femoral head prosthesis was implanted.

Antibiotic prophylaxis was performed within 30 min before incision and the first 24 h postoperatively. Low molecular weight heparin was used for anticoagulation preoperatively, and rivaroxaban was used within 35 days postoperatively. Patients are allowed to non-weight-bearing walking from the second day after surgery. Based on radiological results and subjective experience, affected limbs are allowed to gradually bear weight.

### Radiographic assessment

**Fig. 2:**
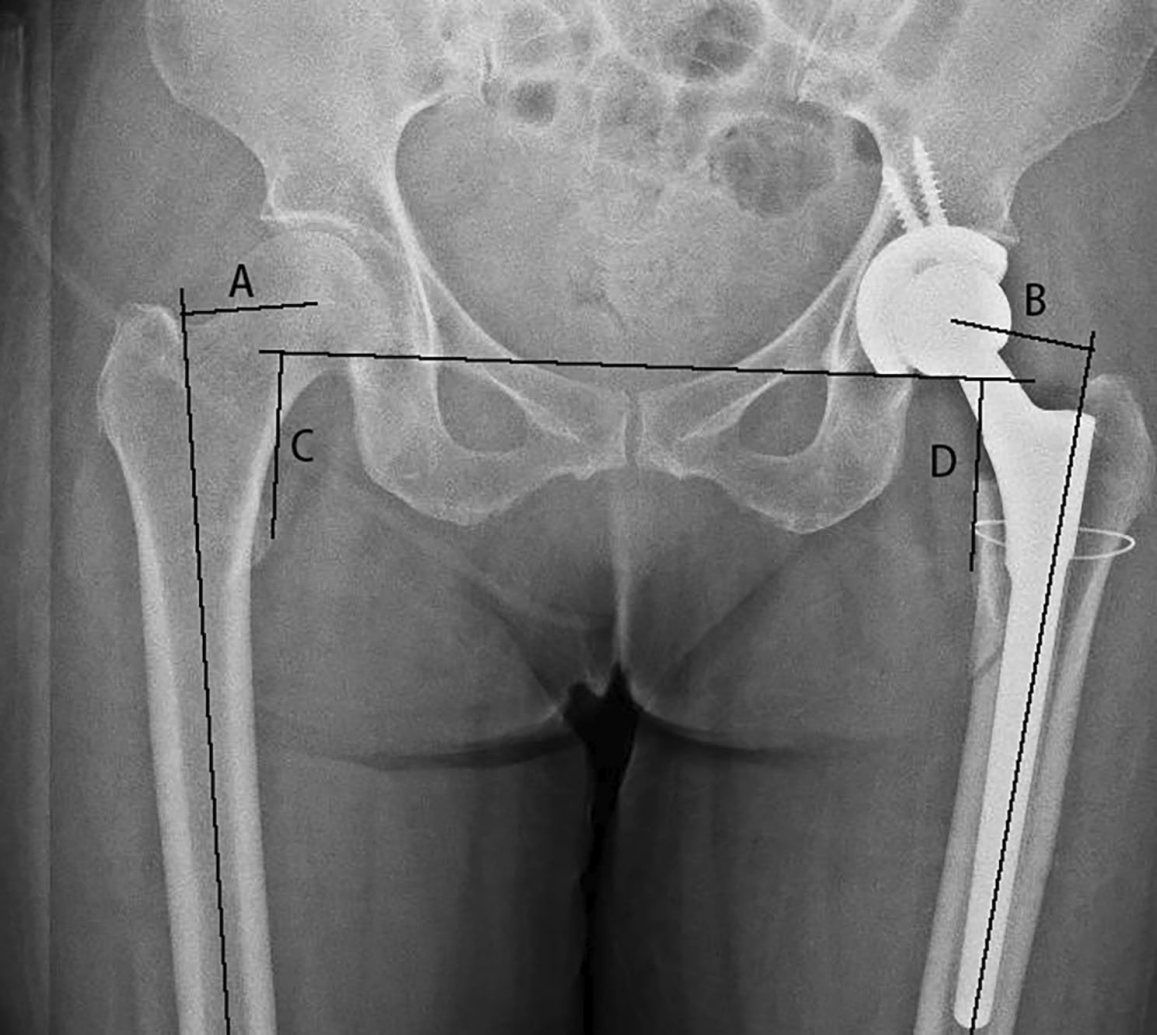
**A**: FO of the healthy side **B**: FO of the affected side **C**, **D**: distance from the line of both teardrops to the center of the lesser trochanter

Method of taking standard orthopantomograms of both hips: The patient is placed in a supine position with both lower limbs extended. The tips of the feet are internally rotated by 15°-20° so that the medial sides of both toes touch each other. The femoral neck was placed in the coronal position. With the superior border of the pubic symphysis as the center of projection, the X-ray is projected vertically, and the distance of projection is 1 m [12].

We measured all X-rays with the same magnification. As Fig. 2 shows, FO was measured with a perpendicular line from the center of the femoral head or femoral head prosthesis to a line representing the anatomic axis of the femur [13]. The length of both limbs was measured as a line from the line of both teardrops to the center of the lesser trochanter [14]. Subsidence was assessed by measuring the vertical migration of the femoral stem as shown in a previous study [15].

According to the difference between the postoperative FO of the affected side and healthy side, those (Group A) whose difference was ≤ 4 mm were considered to have FO reconstruction [16]. According to the difference between the length of the affected limb and healthy limb, those whose difference is ≤ 5 mm are considered to have limbs of equal length.

### Follow‑up

When we followed up patients at 1, 6, and 12 months postoperatively and at their last visit, we assessed the function of the affected hip joint. The HHS score was also calculated, and the range of motion of the hip joint was recorded at 12 months postoperatively and at the last visit. Patients received anteroposterior pelvic and lateral X-rays of the affected hip joint to evaluate fracture healing and to evaluate whether there was femoral stem subsidence or loosening of the prosthesis. During the follow-up, patents returned to the clinic if complications such as dislocation and infection occurred, and immediate treatment was administered.

### Statistical analysis

SPSS 22.0 software was used for all statistical analyses. The measured data are statistically expressed as the mean ± standard deviation. Continuous variables between two groups were compared by t tests. Categorical data were expressed by N (%). The χ2 test or Fisher’s exact probability method was used for group comparisons according to the sample size and number in each cell. P < 0.05 was considered significant.

## Results

### General information

A total of 71 patients with VB2 were reviewed. Of these, 5 patients received conservative treatment due to contraindications for the operation, 2 patients received only open reduction and internal fixation, 4 patients had polytrauma, 10 patients had walking difficulties before fracture, and 8 patients were lost to follow-up. Finally, 42 patients (22 males, 20 females; 69.4 ± 11.0 years) were included in this study. The reasons for primary arthroplasty were femoral neck fracture (20 patients), osteoarthritis of the hip (12 patients) and femoral head necrosis (10 patients). Forty patients received THA, and 2 patients received hemiarthroplasty.

**Fig. 3:**
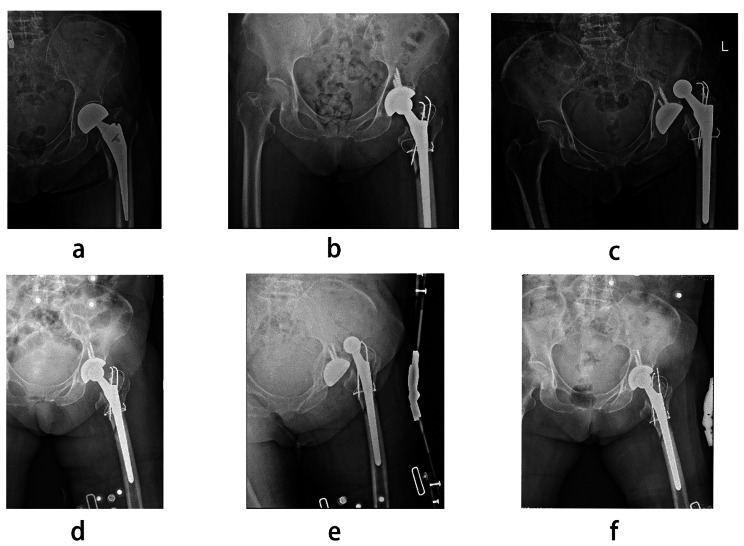
**a** A 76-year-old female who sustained a Vancouver type B2 fracture 21 months after hemiarthroplasty due to femoral neck fracture. **b** The patient underwent revision surgery with a nonmodular prosthesis, but postoperative pelvic anteroposterior X-ray showed that FO was not restored. **c** and **d** The patient had hip joint dislocation 1 month after revision surgery and received closed reduction. **e** and **f** The patient had redislocation of the hip joint 1 week after closed reduction and received closed reduction

**Fig. 4:**
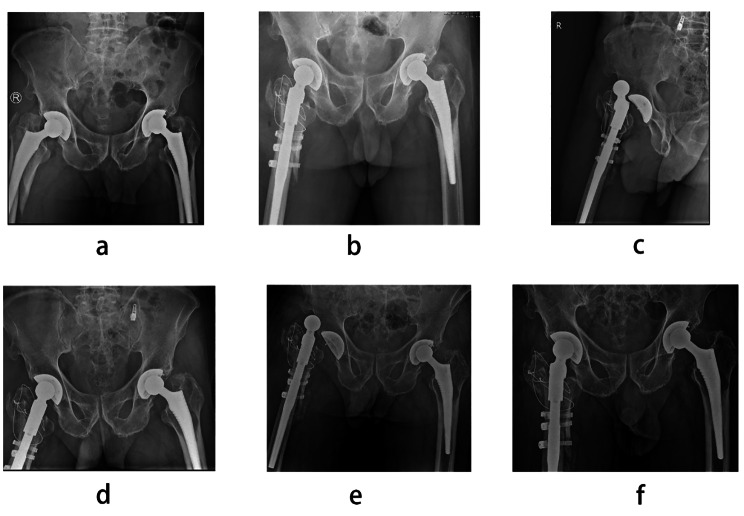
**a** A 73-year-old male who sustained a Vancouver type B2 fracture 2 years after THA due to femoral head necrosis. **b** Patient underwent revision surgery with a modular prosthesis, but postoperative pelvic anteroposterior X-ray showed that FO was not restored. **c** and **d** The patient had hip joint dislocation 1 month after revision surgery and received closed reduction. **e** and **f** The patient had redislocation of the hip joint 2 weeks after closed reduction and received closed reduction

The mean follow-up time of the 42 patients was 34.3 ± 17.3 months. During the follow-up, 1 patient died of cerebrovascular stroke in the fourth year after surgery, and 1 patient had a PPFF of Vancouver C in the affected limb and received open reduction and internal fixation in the third year after surgery. Four patients had hip joint dislocation and received close reduction. Of these, 2 patients had redislocation of the hip joint, and the affected hip joint was restricted by the hip abduction brace after close reduction (Figs. 3 and 4). All patients achieved fracture healing. At the last visit, the FO of the affected limb was 41.36 ± 6.24 mm, and the FO of the healthy side was 40.70 ± 7.14 mm. According to the difference between them, patients were divided into Group A (difference ≤ 4 mm) and Group B (difference>4 mm).

### Comparison of clinical outcomes between Group A and Group B

There was no significant difference in age, sex or BMI between the two groups (P > 0.05, T test). The HHS of Group A was higher than that of Group B at 12 months after surgery and at the last visit (P < 0.05, T test). The proportion of patients with equal limb length in Group A was higher than that in Group B (P < 0.05, χ2 test). The dislocation rate in Group A was lower than that in Group B (P < 0.05, Fisher’s exact probability method) (Table 1).

**Table 1:** Patient demographics and clinical characteristics

Characteristics	Group A (n = 26)	Group B(n = 16)	p value
Age(x ± s, years)	69.5 ± 10.5	69.3 ± 12.0	0.944
Gender (M/F)	14/12	8/8	0.808
BMI(kg/m^2^)	23.6 ± 2.4	23.1 ± 2.1	0.551
HHS (12 months after surgery)	81.5 ± 6.5	76.9 ± 5.9	0.026
HHS(last visit)	86.8 ± 6.1	82.4 ± 5.8	0.028
Equal leg length	22	9	0.042
Dislocation	0	3	0.049

### Comparison of range of motion between Group A and Group B

There was no significant difference in the range of flexion, adduction, internal rotation and external rotation between the two groups at 12 months after surgery (P > 0.05, T test). The range of abduction in Group A was larger than that in Group B at 12 months after surgery (P < 0.05, T test) (Table 2).

**Table 2:** Range of motion of the affected hip joint 12 months after surgery

Motion of joint	Group A (n = 26)	Group B(n = 16)	p value
Flexion	108.3 ± 7.3	106.0 ± 9.6	0.536
Adduction	19.0 ± 2.9	18.6 ± 2.1	0.640
Abduction	30.8 ± 5.6	21.7 ± 4.8	0.000
Internal rotation	22.5 ± 3.4	22.9 ± 3.0	0.720
External rotation	27.6 ± 4.2	26.3 ± 4.2	0.334

There was no significant difference in the range of flexion, adduction, internal rotation and external rotation between the two groups at the last visit (P > 0.05, T test). The range of abduction in Group A was larger than that in Group B at the last visit (P < 0.05, T test). (Table 3).

**Table 3:** Range of motion of the affected hip joint at the last visit

Motion of joint	Group A (n = 26)	Group B(n = 16)	p value
Flexion	115.6 ± 7.1	112.3 ± 9.7	0.216
Adduction	22.9 ± 3.4	22.3 ± 2.9	0.512
Abduction	37.4 ± 5.4	26.6 ± 4.9	0.000
Internal rotation	25.7 ± 3.3	26.6 ± 2.9	0.370
External rotation	32.1 ± 4.1	30.8 ± 4.2	0.342

### Comparison of clinical outcomes between the modular group and nonmodular group

The proportion of patients with FO reconstruction in the modular group was higher than that in the nonmodular group (P < 0.05, χ2 test). The HHS of the modular group at 12 months after surgery was higher than that of the nonmodular group (P < 0.05, T test); however, this difference was not significant at the last visit (P > 0.05, T test). The proportion of patients with equal leg length was higher in the modular group than in the nonmodular group (P < 0.05, χ2 test). For dislocation, there was no significant difference between the two groups (P > 0.05, Fisher’s exact probability method). Compared with the modular group, the nonmodular group tended to have larger subsidence (P < 0.05, T test). (Table 4)

**Table 4:** Clinical characteristics

Characteristics	Modular Group(n = 20)	Nonmodular Group(n = 22)	p value
FO restoration	16	10	0.021
HHS (12 months after surgery)	82.0 ± 5.9	77.8 ± 6.7	0.040
HHS(last visit)	87.0 ± 6.0	83.4 ± 6.2	0.065
Equal leg length	18	13	0.023
Dislocation	1	2	1.000
Subsidence(mm)	1.28 ± 0.98	2.08 ± 1.43	0.045

## Discussion

The length of the FO directly affects the conduction of gravity. When FO is insufficient, the abductors need to be fully contracted to compensate for shortening of the force arm, which may lead to fatigue and limp [17]. Long-term increased stress on the hip joint also increases prosthesis wear. When FO is excessive, lengthening of the force arm increases micromovement of the femoral prosthesis, thus affecting the bone ingrowth and service life of the prosthesis. Restoration of FO is important for restoration of abductor muscle strength and balance of soft tissue tension, maintaining hip joint stability, reducing wear of prosthesis and preventing postoperative dislocation [18, 19]. However, Bourne reported that the proportion of FO restoration in some patients undergoing THA was only approximately 40% [16]. Accurate prereplacement prediction, measurement during replacement and the use of effective prosthetic systems contributed to the restoration of FO. The author found in clinical practice that FO reconstruction was difficult and unsatisfactory in revisions of patients with PPFF, resulting in hip dysfunction, dislocation and leg length discrepancy. This study aimed to investigate the importance of FO reconstruction and prothesis selection in revisions of patients with PPFF.

The FO of the operated side is considered to be restored if the difference in FO between both sides is within 4 mm [16]. Mahmood reported that the WOMAC (Western Ontario and McMaster Universities osteoarthritis index) score decreased in patients whose postoperative FO was smaller than the preoperative measurement, with the strength of the adductor muscle decreasing and the frequency of walking aid use increasing [20]. On the other hand, lengthening of the FO can also lead to some complications, such as pain around the greater trochanter and loosening of the prosthesis. Liebs recruited 362 patients to study the effect of FO on pain after THA. The results showed that patients whose FO decreased after surgery reported less pain than those whose FO increased or changed little [21]. Therefore, insufficient or excessive postoperative FO can have adverse effects on hip function. Age and sex have influence on stems sizes and neck choices in THA, thus affecting FO and hip joint function [22].In our results, there was no significant difference in age, sex or BMI between the two groups. The HHS of Group A was higher than that of Group B at 12 months after surgery and at the last visit, which proved that restoring FO helped restore the function of the hip joint.

If FO decreases, the femur will be close to the pelvis, which may easily lead to the limitation of the range of motion of the hip joint and the relaxation of the surrounding soft tissues, resulting in instability and postoperative dislocation of the hip joint [9].

In our results, the dislocation rate in Group A was lower than that in Group B. Figures 3 and 4 show that two patients developed recurrent dislocation due to insufficient FO. Revision might cause much damage, so patients were asked to wear hip abduction braces and have long-term bed rest. After fibrous tissue was formed, none of them had dislocation again. Our results also showed that the proportion of patients with equal limb length in Group A was higher than that in Group B. We found one situation was that surgeons chose a prosthesis of improper neck length, resulting in excessive FO with lengthening of the limb or insufficient FO with shortening of the limb. The other situation was that surgeons chose a prosthesis with a smaller FO and a larger collodiaphyseal angle, which lead to lengthening of the limb. In both situations, LLD is related to failure of FO restoration. However, Stijn found no significant differences in LLD between restored FO subgroups and unrestored FO subgroups [23]. Therefore, the relationship between FO restoration and LLD needs further research.

Restoration or lengthening of the FO, which moves the femur outward, reduces its impact on the pelvis, improves the surrounding soft tissue tension, makes the hip joint more stable, and increases the range of motion of the hip joint [24]. In our results, the range of abduction in Group A was larger than that in Group B 12 months after surgery and at the last visit, and there was no significant difference in other directions. The results showed that FO restoration increased the range of abduction, thus improving the function of the hip joint.

We found that in revisions of patients with periprosthetic fractures, surgeons should implant the prosthesis in the distal femoral medullary cavity first, rather than reducing and fixing the fracture before reaming the femoral canal and implanting the revision femoral prosthesis. The latter method may result in redisplacement of the fracture during the reaming process. As a result, the prosthesis may achieve stable fixation with the proximal fracture block but not with the distal fracture end, thus resulting in loosening. After implanting the prosthesis and reducing and fixing the fracture, surgeons may need to adjust the length, FO and anteversion angle of the prosthesis to avoid LLD and dislocation. Compared with nonmodular prostheses, modular prostheses can meet the needs of distal and proximal medullary cavity differences and can be adjusted in length, FO and anteversion angle, reducing the difficulty of surgical operation [25, 26]. In addition, the distal portion of the prosthesis is well matched to the medullary cavity, thus leading to better axial and rotational stability and requiring less length of interface fixation than a single stem prosthesis. Weiss et al. examined 90 revision surgeries with MP tapered fluted modular titanium stems, and the retention rate of the prosthesis at 5 years was 98% [27]. Joshua reported that the survivorship of tapered fluted modular stems free of reoperation or implant revision at 5 years was 89% and 93%, respectively [28]. There are few reports about the difference between modular and nonmodular prostheses in revisions of patients with PPFF. In our results, the proportion of patients with FO reconstruction in the modular group was higher than that in the nonmodular group, which suggests that modular prostheses have advantages in restoring FO in revisions of patients with PPFF. A higher proportion of FO reconstruction also resulted in a higher HHS at 12 months after surgery.

Due to the fracture of the proximal femur and loss of anatomical structure, it is difficult to control the anteversion angle and length of the neck during revision surgery, especially in the nonmodular group, which can easily lead to postoperative hip dislocation. Park [29] reported 27 patients with Vancouver type B2 or B3 fractures who underwent revision surgery with an MP prosthesis, and no postoperative hip dislocation occurred. In our results, the proportion of patients with dislocation was lower in the modular group, but the difference was not significant, which may be due to the small number of cases. To prevent postoperative hip dislocation, we should reconstruct the posterior rotator muscle group and repair the posterior articular capsule to maintain soft tissue balance around the prosthesis. Moreover, we can take full advantage of the modular prosthesis and use proximal prostheses and femoral heads of different sizes for repetitive adjustment to restore FO and obtain joint stability. In our results, the proportion of patients with equal leg length was higher in the modular group than in the nonmodular group. This is because we can adjust the length of the limb by using components of different lengths in the modular group. Our results also showed that the subsidence of the modular group was smaller than that of the nonmodular group. This may be attributed to the tapered fluted design, which facilitates anti-rotation and adequate contact between the distal end of the prosthesis and the femur. In addition, the 3-degree curvature of the modular stem helped accommodate the bow of the patient’s femur and achieve sufficient canal filling [30]. In addition to the above advantages, modular tapered fluted stems for femoral revision show excellent outcomes in patients with Paprosky 3, 4 femoral defects [31], so modular stems may be a good choice for revisions in patients with extensive femoral bone loss.

The present study has several limitations. First, the retrospective design used herein has inherent restrictions. Second, the revisions were performed by two groups of surgeons, which might have led to bias in the outcome. Third, the number of patients was small, and there may be some bias in the results. Fourth, there are few types of prothesis and the conclusion needs to be proved by more types of prothesis.

## Conclusion

Restoration of FO can improve the function of the hip joint and prognosis in revisions of patients with VB2. Surgeons should restore FO and avoid LLD and dislocation as much as possible. Modular prostheses have advantages in FO restoration and reduce the occurrence of complications, which may be a good option for revisions in patients with PPFF.

## Data Availability

The datasets generated and analyzed during the current study are not publicly available due to limitations of ethical approval involving the patient data and anonymity but are available from the first author (Lei Sun) on reasonable request.

## Abbreviations

FO: Femoral offset
HHS: Harris Hip Score
LLD: Limb length discrepancy
PPFF: Periprosthetic femoral fracture
